# Supplementary material for: Mapping functional diversity from remotely sensed morphological and physiological forest traits
Source: Nat Commun. 2017 Nov 13;8:1441. doi: 10.1038/s41467-017-01530-3 (PMC5682291; doi:10.1038/s41467-017-01530-3)
Supplement: Supplementary file 1 — Supplementary Information [file 41467_2017_1530_MOESM1_ESM.pdf]

Supplementary Information

Supplementary Note 1. Simulating the radiative budget using the 3D radiative transfer model DART

Radiation was simulated as incoming photosynthetically active radiation (PAR) in the range of 400 - 700 nm at top of canopy. The simulation was performed using the 3D coupled canopy-atmosphere radiative transfer model DART[1] according to clear sky atmospheric conditions for a rural aerosol model with a visibility of 23 km. The whole Laegern forest was represented as 3D voxel grid with 2 m voxel side length in DART following the parametrisation of Schneider and colleagues[2]. Mean daily incoming radiation of 2010 was approximated by deriving the total amount of incoming radiation for 8 days during the year, of which each day was simulated with nine sun angles and a 4th order polynomial to integrate the radiation over the whole day. A comparison to the fluxtower based on global radiation on 26 June 2010 shows a good agreement between simulated and measured values (Supplementary Fig. 13).

Supplementary Note 2. Simulating canopy spectra using the 3D radiative transfer model DART

To assess the applicability of spectral indices from the leaf to the canopy level, we modelled canopy spectra with the 3D radiative transfer model DART[1]. The DART model was parametrised following Schneider and colleagues[2] with a 3D description of the forest canopy at Laegern based on airborne laser scanning data. A scene of 400 x 400 m was simulated to cover the 5.5 ha Laegern core site and a buffer area to avoid any border effects at the edges of the site. The simulations were performed with 2 m spatial resolution based on the spectral band definitions, illumination and viewing geometry of the two imaging spectrometer acquisitions on 26 and 29 June 2010. The simulated ortho-images of canopy reflectance were combined and aggregated to 6 m spatial resolution to reduce shadow effects, following the same approach as applied to the imaging spectrometer data (see Methods of main manuscript).

References

1. Gastellu-Etchegorry, J.-P. *et al.* Discrete anisotropic radiative transfer (dart 5) for modeling airborne and satellite spectroradiometer and lidar acquisitions of natural and urban landscapes. *Remote Sensing* **7**, 1667–1701 (2015).

2. Schneider, F. D. *et al.* Simulating imaging spectrometer data: 3d forest modeling based on lidar and in situ data. *Remote Sensing of Environment* **152**, 235–250 (2014).

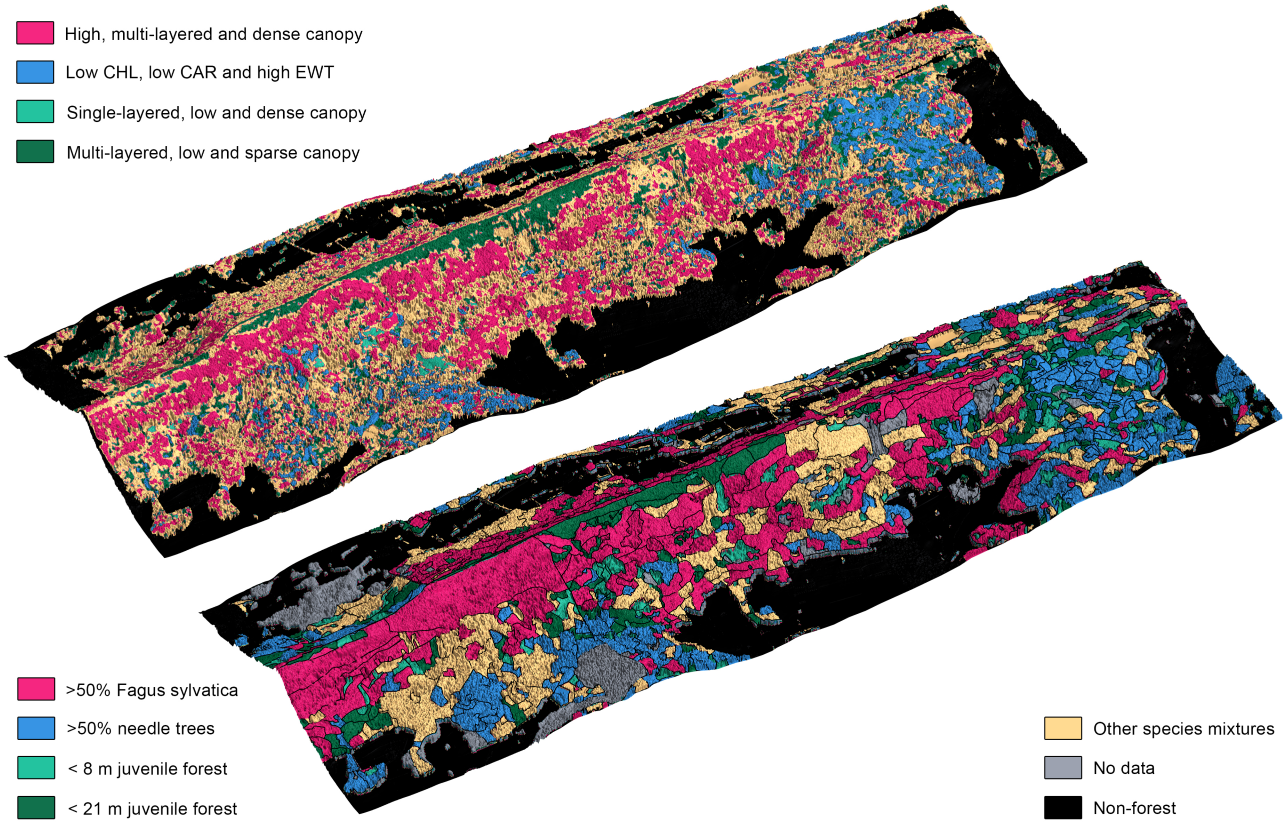

Supplementary Figure 1: Maps showing trait classes based on remotely sensed morphological and physiological traits and stand polygons of the state government (Kt. AG + ZH). The trait classes shown here in pink, turquoise and green are based on canopy height (CH), layering (FHD) and density (PAI), whereas the class shown in blue is based on chlorophyll (CHL), carotenoids (CAR) and equivalent water thickness (EWT).

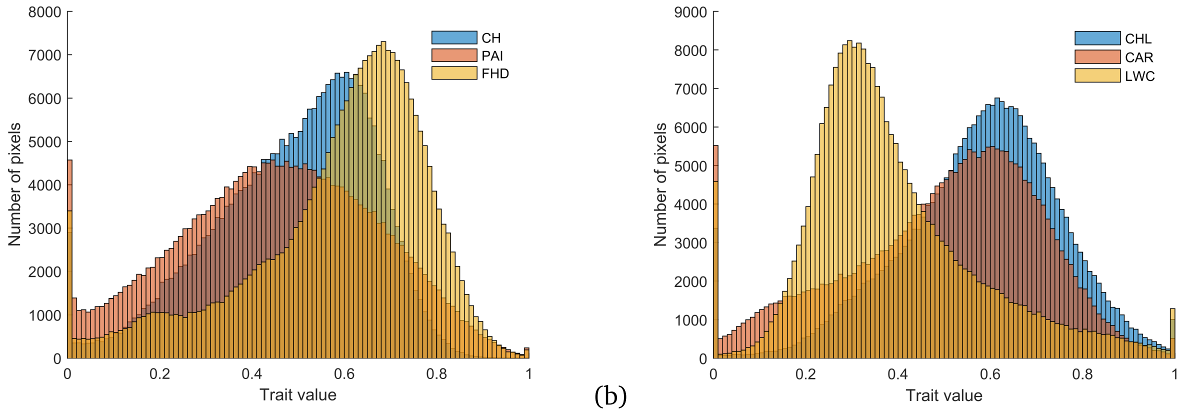

Supplementary Figure 2: Frequency distributions of (a) morphological traits canopy height (CH), plant area index (PAI), and foliage height diversity (FHD) and (b) physiological traits chlorophyll (CHL), carotenoids (CAR), and equivalent water thickness (EWT).

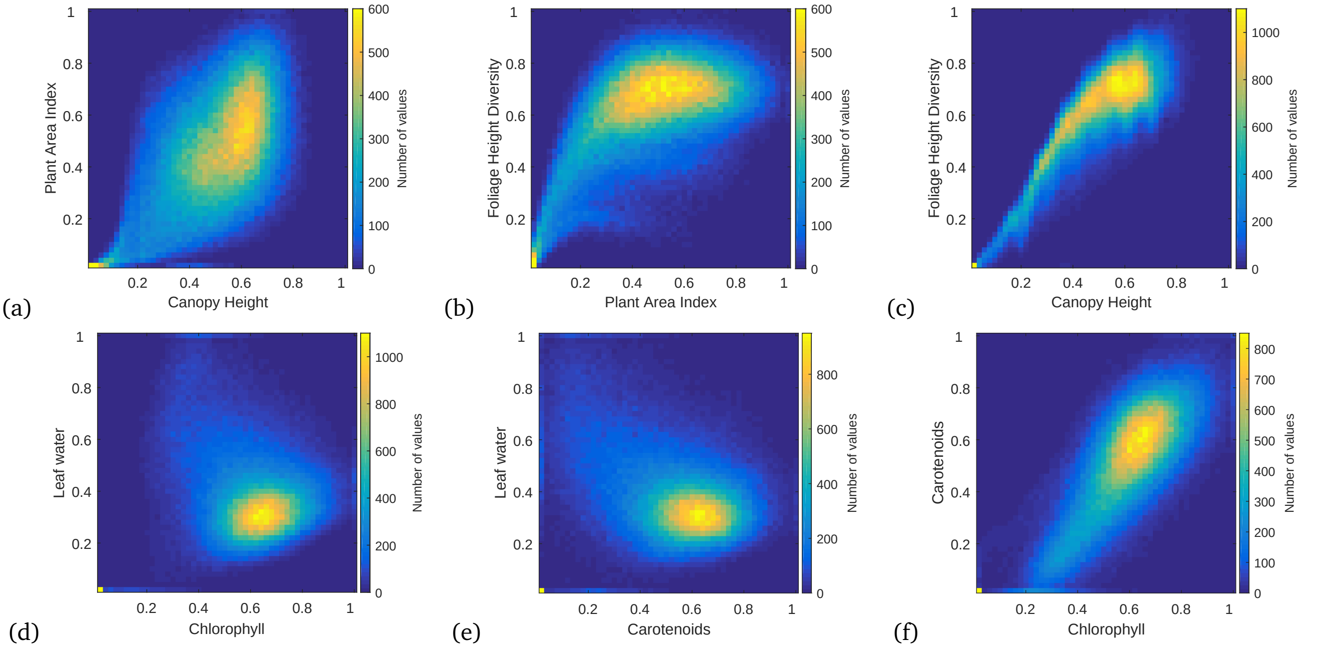

Supplementary Figure 3: Trait correlations among the morphological traits plant area index, canopy height and foliage height diversity (a-e) and among the physiological traits leaf water, chlorophyll and carotenoids (d-f).

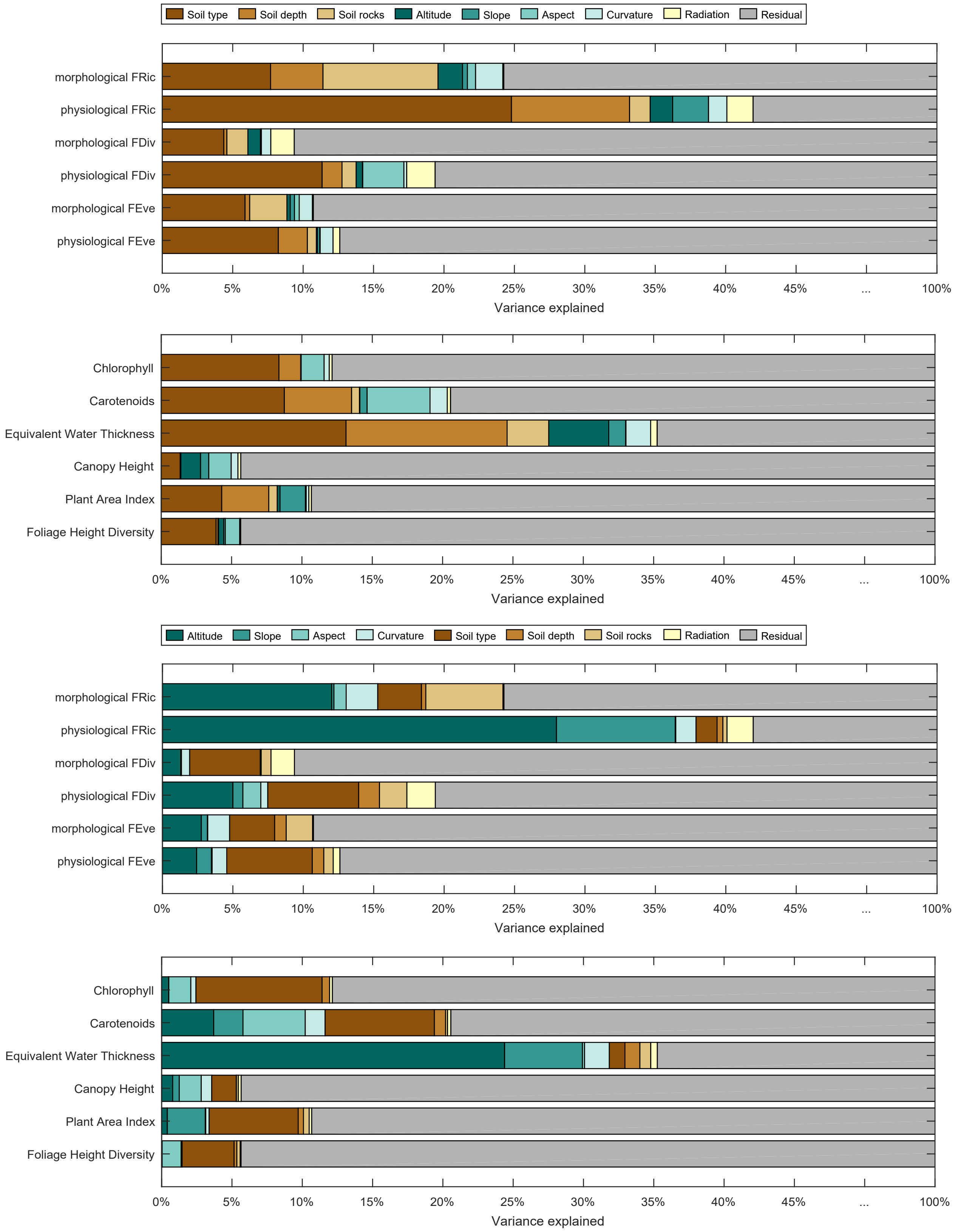

Supplementary Figure 4: Amount of variance in functional diversity and functional traits explained by soil (soil type, soil depth, amount of rocky material), topography (altitude, slope, aspect, curvature) and radiation (mean daily photosynthetically active radiation). The order of the legend corresponds to the order in the ANOVA type I, with soil variables first (upper panel) and topographic variables first (lower panel). The independent variables used in the ANOVA are shown in Supplementary Fig. 5

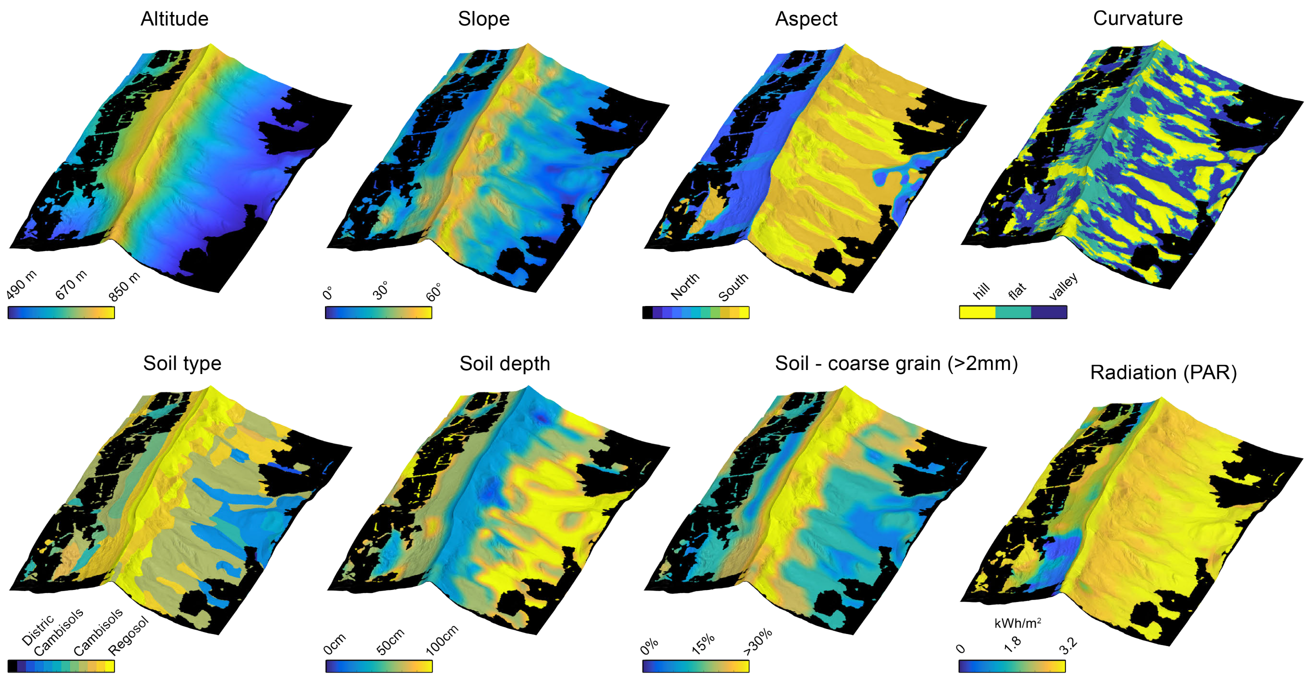

Supplementary Figure 5: Environmental variables covering the western part of the study area. Altitude, slope, soil depth, amount of coarse grain material in the soil and radiation (mean daily PAR, see Supplementary Note 1) are continuous variables, averaged at 60 m radius for use in an ANOVA (see Statistical analysis in Methods of main manuscript). Aspect, curvature and soil type are categorical variables. Soil type consists of 8 classes, whereas Distric Cambisols (blue), Cambisols (olive) and Regosol (yellow) are among the most abundant soils.

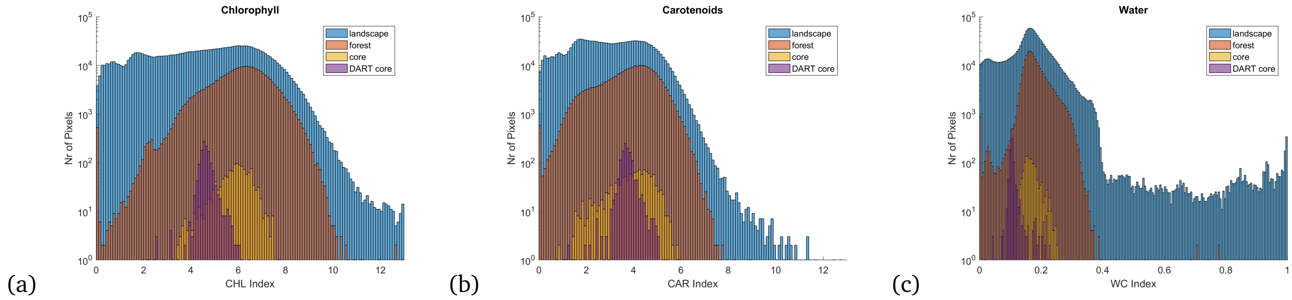

Supplementary Figure 6: Observed trait ranges derived from imaging spectroscopy at landscape level (including agricultural fields), at forest level (whole Laegern), and at a core site of 1307 trees on 5.5 ha for (a) chlorophyll, (b) carotenoids, and (c) equivalent water thickness. The figures show a comparison to modelled trait ranges at the core site using the 3D radiative transfer model DART. The modelled ranges are narrower since constant leaf optical properties were used for broadleaf and needle trees.

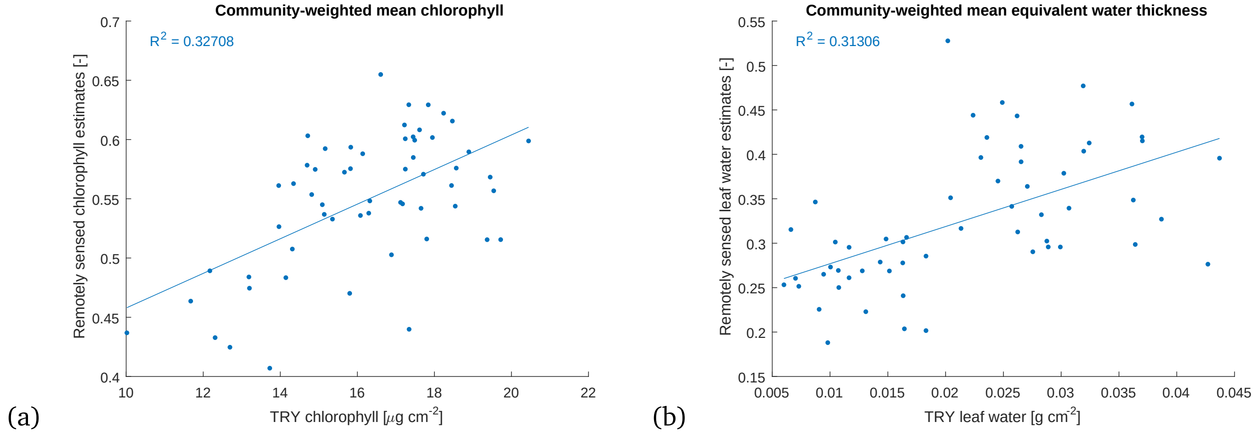

Supplementary Figure 7: Community-weighted mean (a) chlorophyll and (b) equivalent water thickness of 13 deciduous broadleaf and evergreen coniferous tree species at the 5.5 ha core site compared to trait values calculated based on the functional trait database TRY.

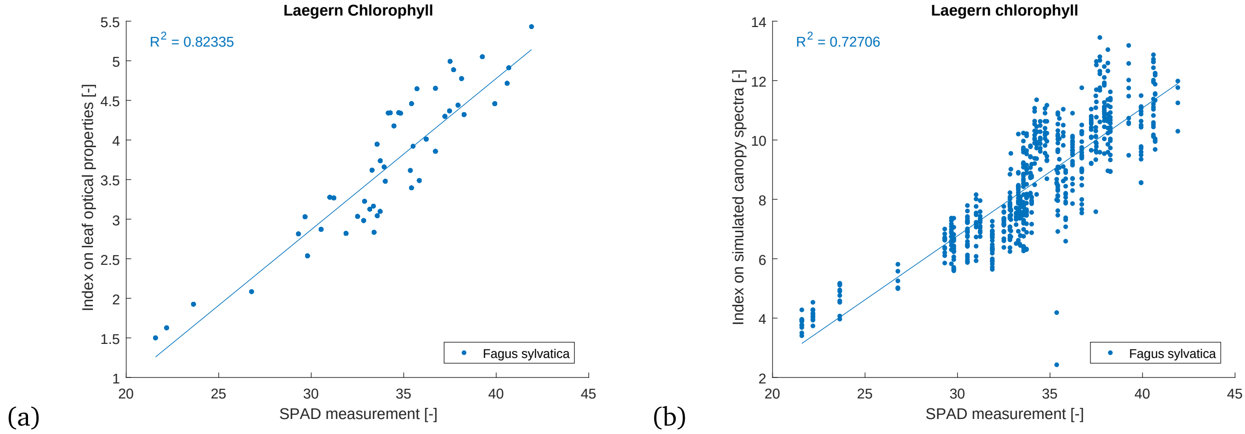

Supplementary Figure 8: SPAD measurements of 50 *Fagus sylvatica* trees at the 5.5 ha core site compared to corresponding chlorophyll estimates as derived applying a spectral index on (a) leaf optical properties and (b) modelled canopy spectra.

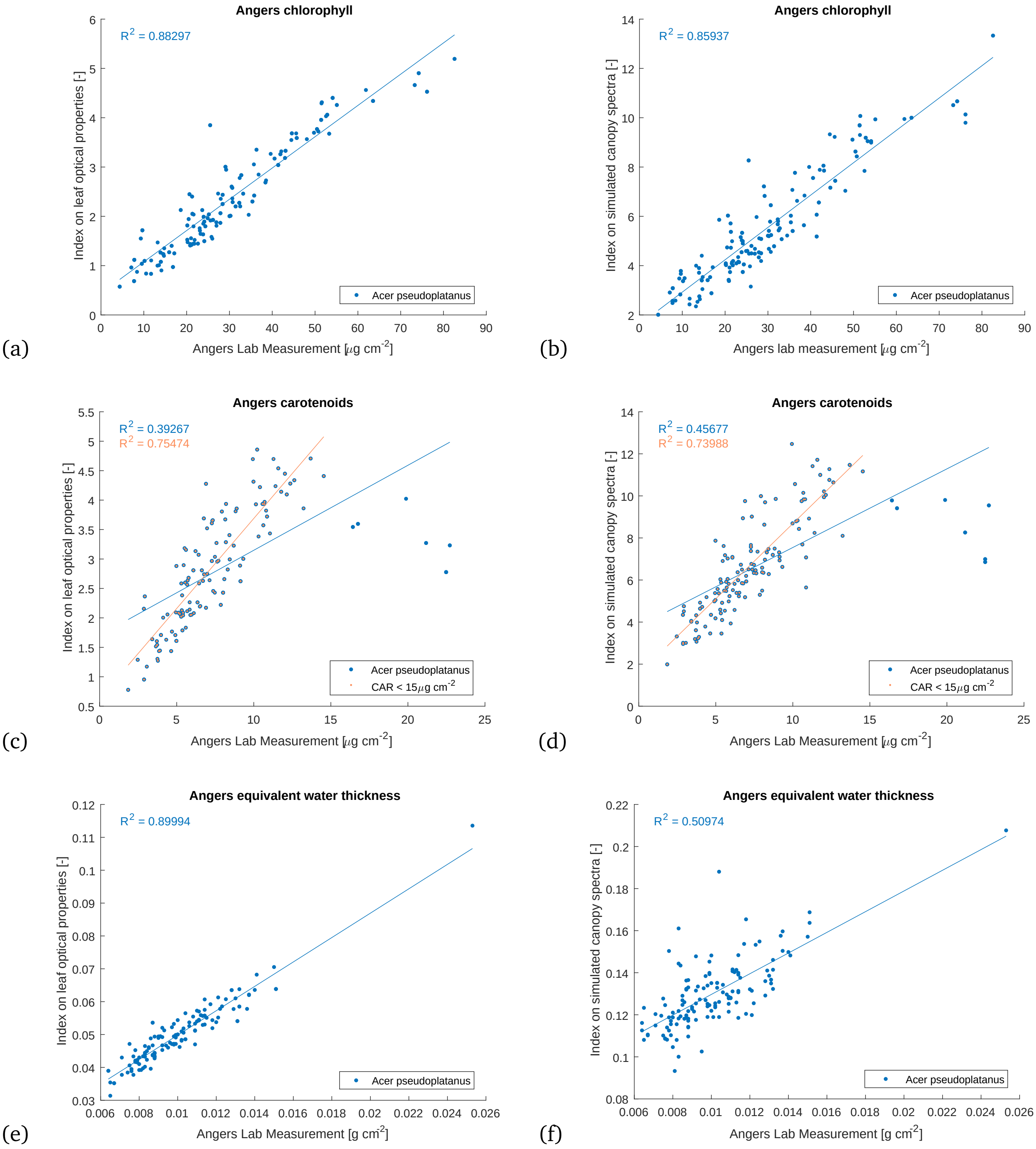

Supplementary Figure 9: Lab measurements of 168 *Acer pseudoplatanus* trees from the ANGERS database of (a-b) chlorophyll, (c-d) carotenoids, and (e-f) equivalent water thickness compared to corresponding trait estimates as derived applying spectral indices on (a,c,e) leaf optical properties and (b,d,f) modelled canopy spectra. Since very high carotenoids values are unlikely to appear at the Laegern forest in summer, a second linear regression was fitted for values below 15 $\mu g\ cm^{-2}$ (orange line, c-d).

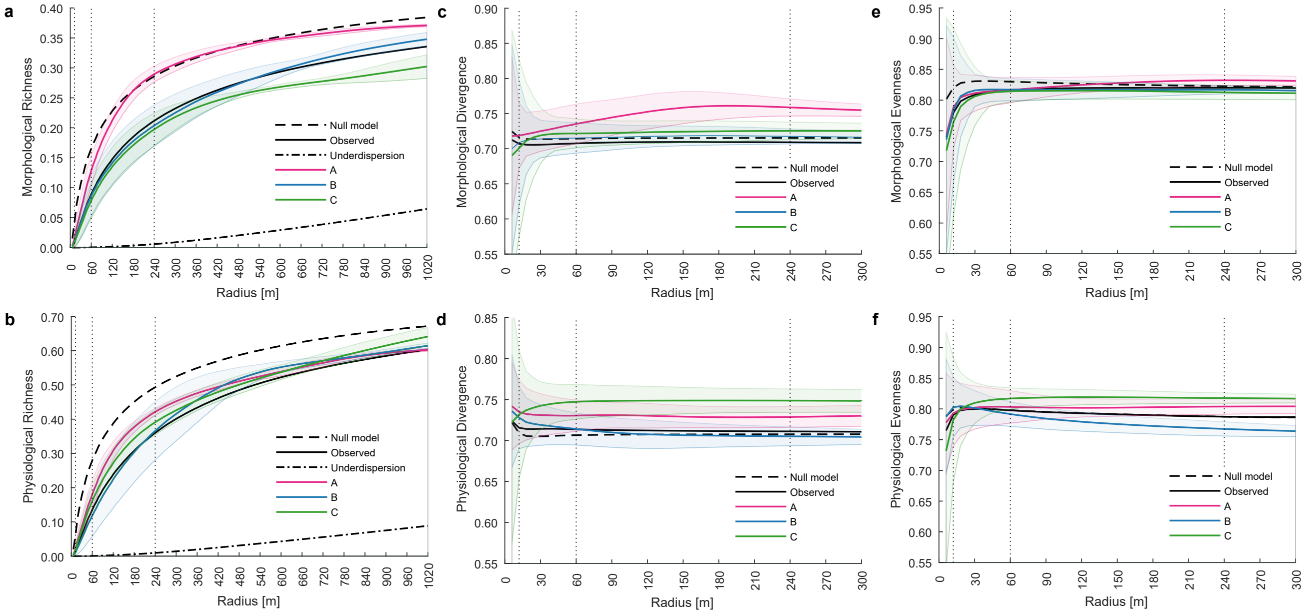

Supplementary Figure 10: Scale dependency of the three functional diversity measures for morphological and physiological traits. Functional (a,b) richness, (c,d) divergence, and (e,f) evenness as a function of radius (diversity-area) for (a,c,e) morphological and (b,d,f) physiological traits. Coloured solid lines A, B, C and coloured areas correspond to mean and standard deviation of subregions A, B, C. Vertical dotted lines correspond to radii as in Fig. 8 (main manuscript).

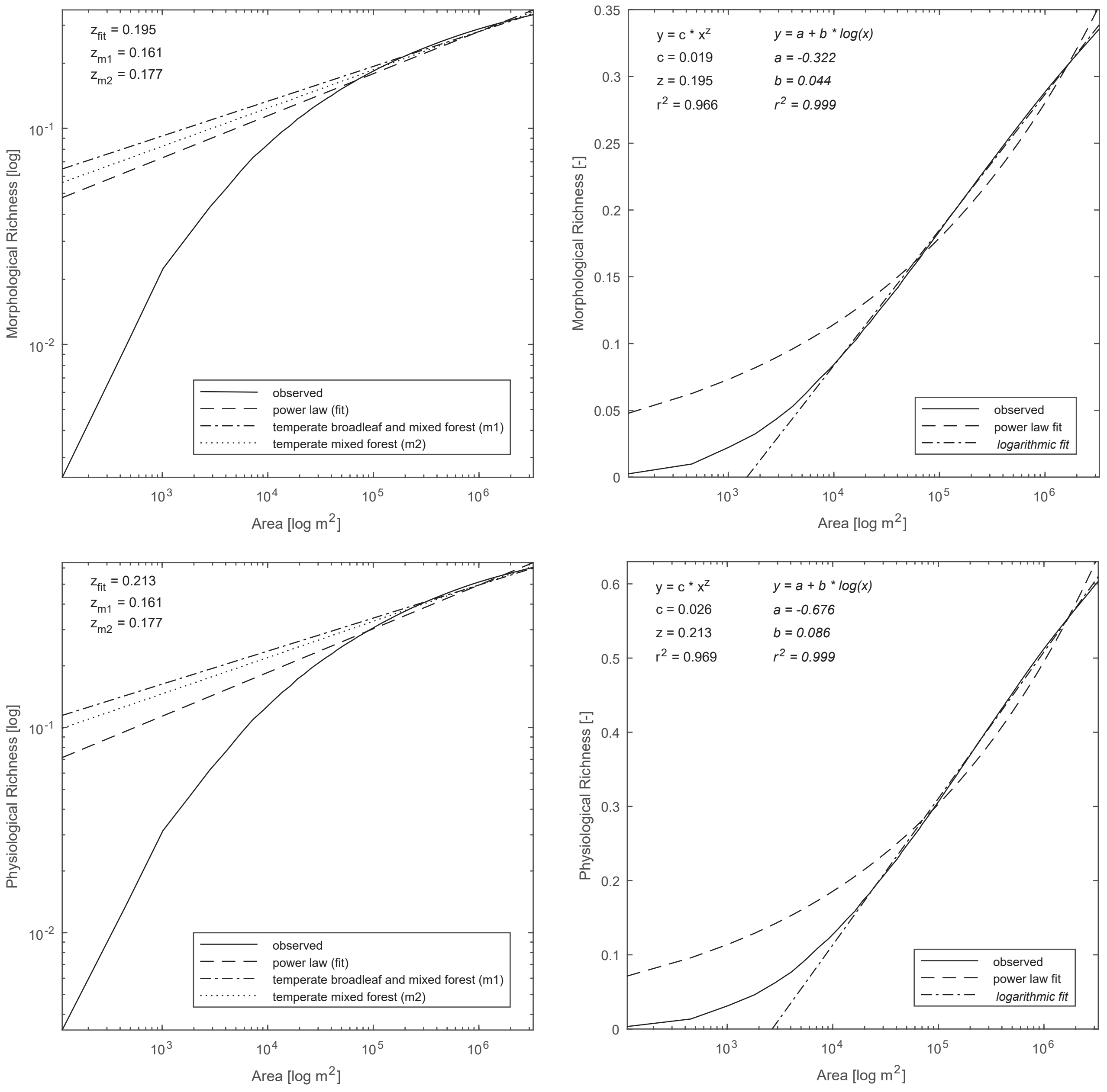

Supplementary Figure 11: Morphological (upper panels) and physiological (lower panels) functional richness-area relationships. Left panels are in log-log scale and show the fit of a power law function (dashed line) to the observed relationship based on the ecosystem mean (solid line) in comparison to two large scale species richness-area relationships based on global models of Gerstner et al. 2014. Right panels show the fit of a power law (dashed line) and a logarithmic (dash-dotted line) function in log-area scale. The logarithmic function has been fitted to areas above 10^4 m^2, where the observed mean values are linear in log-area scale.

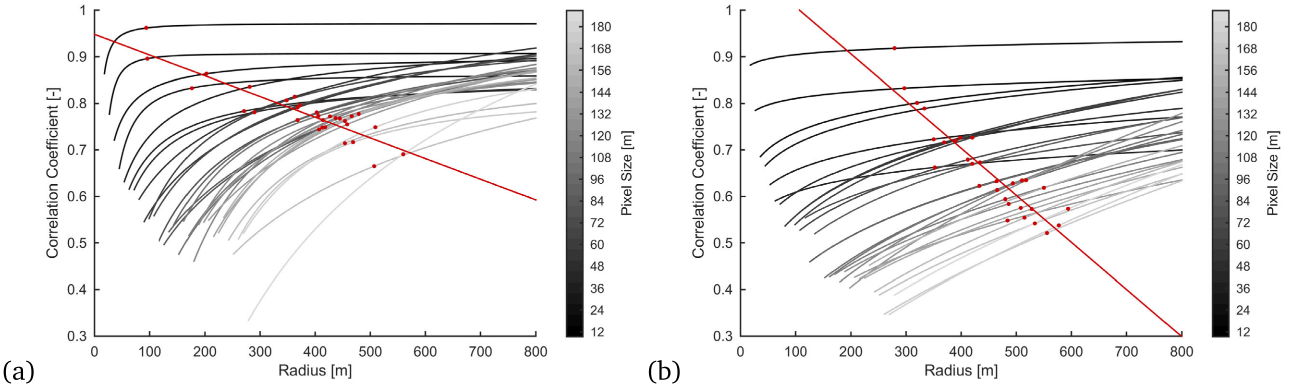

Supplementary Figure 12: Correlation between functional richness patterns of high spatial resolution (6 m pixel size) and increasing pixel size at a given neighbourhood radius for (a) morphological and (b) physiological traits. The red line indicates a linear fit to the knees (red dots) of the curves.

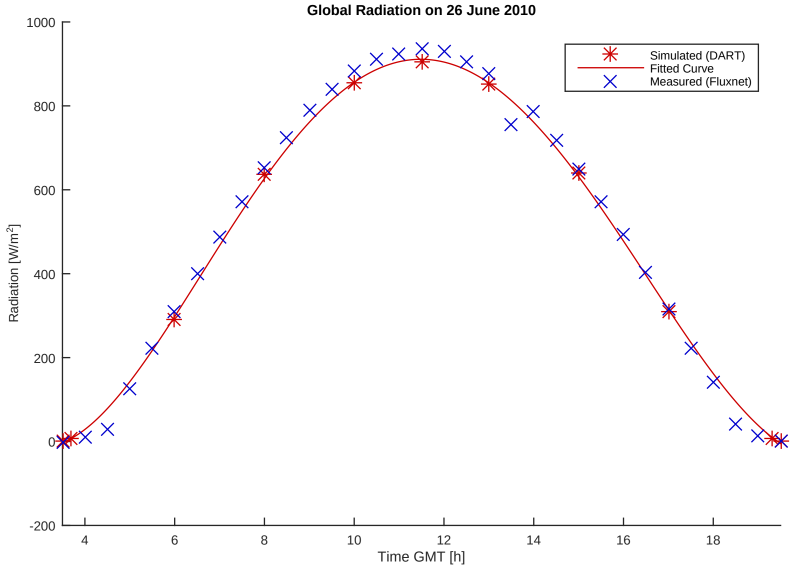

Supplementary Figure 13: Diurnal global radiation as simulated using the DART model and measured on a fluxtower of FLUXNET (CH-Lae).

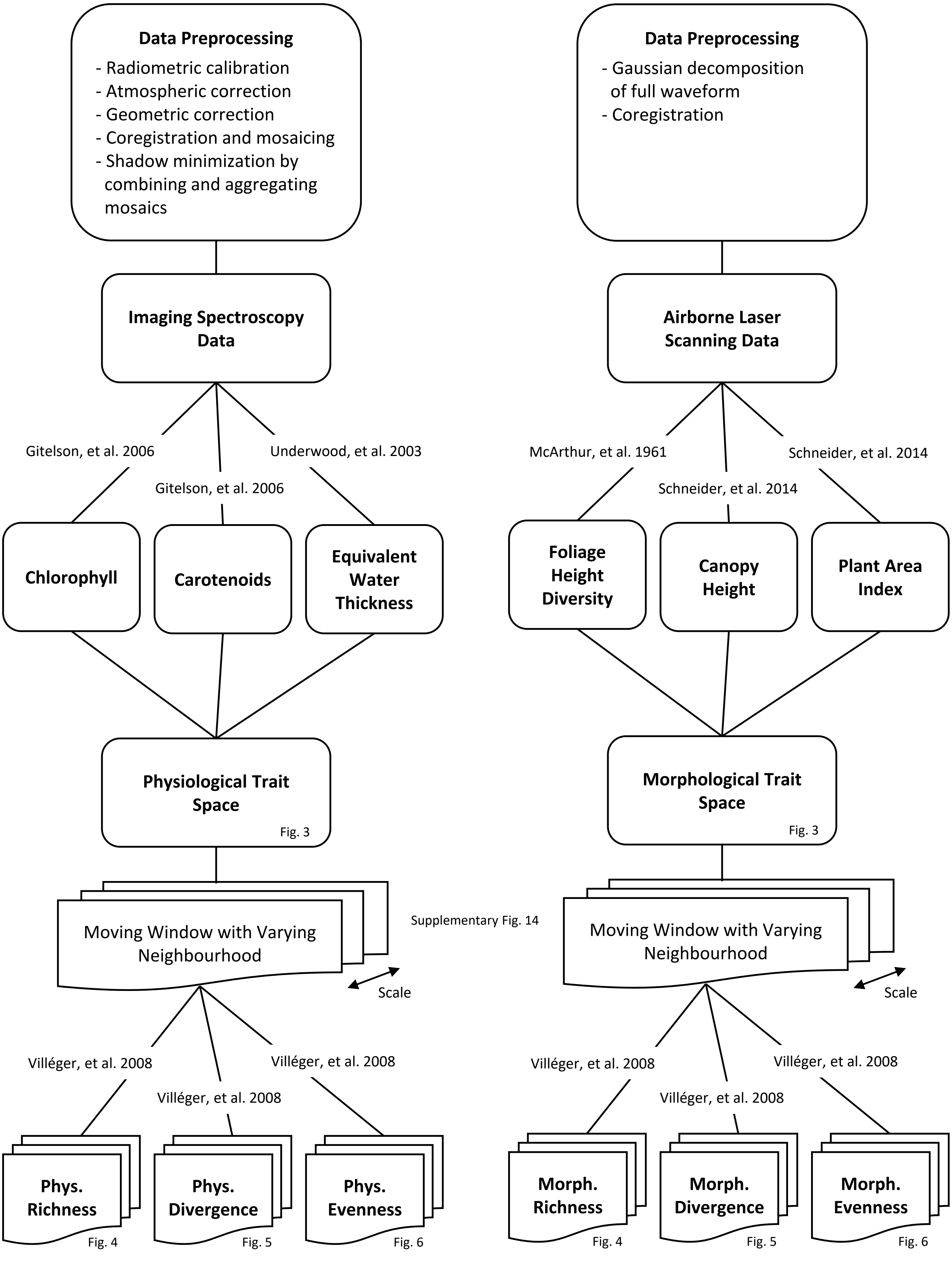

Supplementary Figure 14: Flowchart visualising the work-flow from remote sensing data to physiological (left) and morphological (right) diversity measures. The functional traits are combined to a three-dimensional trait space. By iterating through the pixels using a moving window approach and changing the extent of the neighbourhood, functional diversity measures can be calculated for many scales.

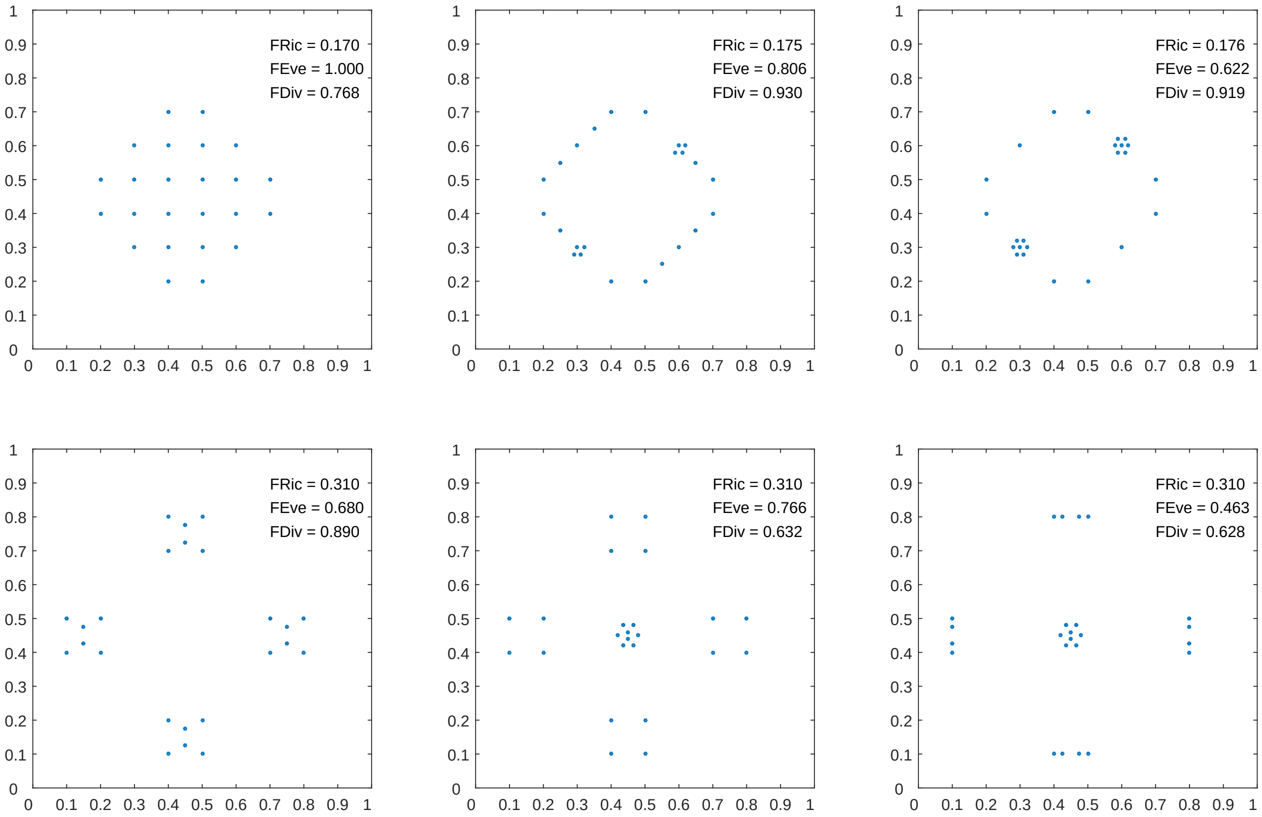

Supplementary Figure 15: Visualization of the three functional diversity indices functional richness, functional evenness and functional divergence.

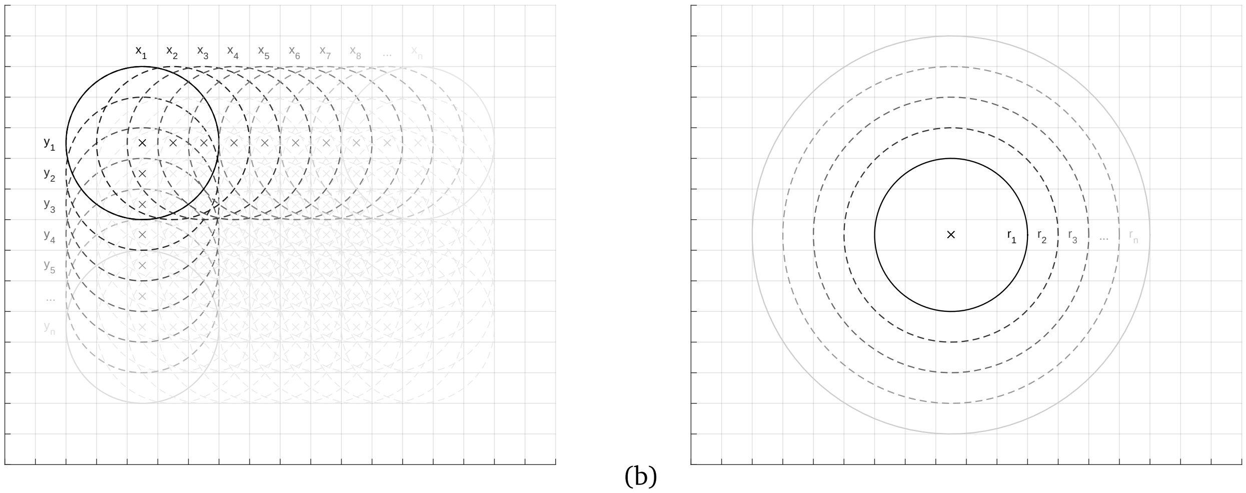

(a) (b)

Supplementary Figure 16: Schematic illustration of (a) the moving window approach and (b) increasing extent. Every pixel $p_{x_{1..n},y_{1..n}}$ is assigned the diversity value calculated based on the radial neighbourhood area with radius $r_{1..n}$.

Supplementary Table 1: ANOVA type I results for functional richness (FRic), divergence (FDiv) and evenness (FEve) explained by topographic variables. The order of the independent variables has been determined by the significance of the variables when tested individually, whereas the most significant are used first in the combined model. The variable 'Aspect' includes the three factors north, south, and flat slopes, whereas 'Curvature' is grouped in valley, ridge, and straight areas. 'Altitude' and 'Slope' are continuous variables. Stars indicate significance levels ***0.001, **0.01 and *0.05.

Dependent Variable	Variable	SumSq	DF	MeanSq	F	pValue	r^2
Morphological FRic	Altitude	0.024352	1	0.024352	48.3763	***1.214e-11	0.246
	Curvature	0.003841	2	0.0019205	3.8152	*0.02273	
	Slope	0.000113	1	0.000113	0.2251	0.63543	
	Aspect	0.001981	2	0.0009905	1.9676	0.14097	
	Error	0.231557	460	0.0005034			
Physiological FRic	Slope	0.16622	1	0.16622	121.4982	***< 2.2e-16	0.71395
	Altitude	0.02802	1	0.02802	20.4843	***7.665e-06	
	Aspect	0.00260	2	0.001299	0.9495	0.3877	
	Curvature	0.01070	2	0.005350	3.9104	*0.0207	
	Error	0.62931	460	0.001368			
Morphological FDiv	Altitude	0.004961	1	0.004961	8.4499	**0.003827	0.034
	Curvature	0.000625	2	0.0003125	0.5322	0.587650	
	Aspect	0.001408	2	0.0007040	1.1991	0.302397	
	Slope	0.000060	1	0.000060	0.1021	0.749410	
	Error	0.270054	460	0.0005871			
Physiological FDiv	Slope	0.010496	1	0.010496	23.4252	***1.776e-06	0.169
	Aspect	0.001859	2	0.0009294	2.0743	0.1268	
	Altitude	0.000019	1	0.000019	0.0415	0.8386	
	Curvature	0.000509	2	0.0002545	0.5680	0.5671	
	Error	0.206111	460	0.0004481			
Morphological FEve	Altitude	0.004251	1	0.004251	13.9823	***0.0002078	0.054
	Curvature	0.002039	2	0.0010196	3.3537	*0.0358118	
	Aspect	0.000703	2	0.0003515	1.1563	0.3155759	
	Slope	0.000194	1	0.000194	0.6396	0.4242760	
	Error	0.139854	460	0.0003040			
Physiological FEve	Slope	0.005621	1	0.005621	14.8399	***0.0001336	0.130
	Aspect	0.000844	2	0.0004218	1.1136	0.3292436	
	Altitude	0.000249	1	0.000249	0.6580	0.4177012	
	Curvature	0.001997	2	0.0009987	2.6364	0.0726994	
	Error	0.174246	460	0.0003788			
